# Bacterial culture and antimicrobial susceptibility results from bovine milk samples submitted to four veterinary diagnostic laboratories in Australia from 2015 to 2019

**DOI:** 10.3389/fvets.2023.1232048

**Published:** 2023-08-10

**Authors:** Charlotte Langhorne, Suman Das Gupta, Sara Horsman, Caitlin Wood, Benjamin J. Wood, Leslie Barker, Ania Deutscher, Rochelle Price, Michael R. McGowan, Mark Humphris, Shahab Ranjbar, Joerg Henning, Justine S. Gibson

**Affiliations:** ^1^School of Veterinary Science, The University of Queensland, Gatton, QLD, Australia; ^2^Gulbali Institute, Charles Sturt University, Wagga Wagga, NSW, Australia; ^3^Department of Agriculture and Fisheries, Biosecurity Sciences Laboratory, Coopers Plains, QLD, Australia; ^4^NSW Department Primary Industries, Elizabeth Macarthur Agricultural Institute, Menangle, NSW, Australia; ^5^The Milk Road, Newry, VIC, Australia

**Keywords:** antibiotic, antimicrobial susceptibility, mastitis, *Streptococcus uberis*, *Staphylococcus aureus*

## Abstract

A 5-year retrospective study was conducted to describe the mastitis-causing organisms isolated from bovine milk samples submitted to four veterinary diagnostic laboratories in Australia. The aim of this study was to identify temporal, geographical, and seasonal patterns of occurrence for the organisms and report the *in vitro* susceptibility of the most common mastitis-causing pathogens. In total, 22,102 milk samples were submitted between 2015 and 2019. The results were reported as positive growth for at least one significant organism (*n* = 11,407; 51.6%), no growth (*n* = 5,782; 26.2%), and mixed/contaminated growth (*n* = 4,913; 22.2%). Culture results for no growth, gram-negative bacteria, and eukaryotic organisms were combined for each region, and they were accounted for between 23 and 46% of submissions. These results represent a subset of mastitis cases for which the antibiotic treatment may not be warranted. A total of 11,907 isolates were cultured from 11,407 milk samples. The most common isolated organisms were *Streptococcus uberis* [41.3%; 95% confidence interval (CI): 40.4–42.1%] and *Staphylococcus aureus* (23.6%; 95% CI: 22.8–24.3%). For *S. uberis* and *S. aureus*, there was an association between a positive culture result and the dairy region. All regions except for the Sub-tropical Dairy region were more likely to culture *S. uberis* compared to the reference, Dairy NSW (*P* < 0.001). Similarly, for *S. aureus*, a positive culture result was more likely in all other dairy regions compared to Dairy NSW (*P* < 0.001). The LISA cluster analysis identified differences between High-High (hotspot) postcodes for *S*. *aureus* and *S*. *uberis* throughout all the analyzed dairy regions. These results highlight the need for further investigations into specific risk factors, such as environmental factors and herd-level predictors, which may have influenced the observed regional variations. Common mastitis-causing pathogens showed overall good susceptibility to a range of antimicrobials used in the treatment of mastitis. On-going surveillance of mastitis-causing pathogens and their antimicrobial susceptibilities will facilitate targeted mastitis control and treatment programs.

## 1. Introduction

Mastitis is a challenging disease of dairy cows worldwide with impacts on animal health and welfare, farm productivity, and profitability (1, 2). In Australia, the bacterial cause of a case of bovine mastitis is generally not diagnosed prior to antimicrobial treatment. However, veterinarians and farmers will have a general idea of the common pathogens and base treatment decisions on the outcome of previous cases and experience. Consequently, it is important to update our understanding of the common mastitis-causing pathogens and their antimicrobial susceptibilities in Australia's dairy regions.

A recent Australian survey of bovine mastitis-causing organisms conducted in 2011–2012 identified *Streptococcus uberis, Staphylococcus aureus, Escherichia coli, Streptococcus dysgalactiae*, and *Corynebacterium bovis* as the most common bacteria cultured from subclinical and clinical mastitis (3). In contrast, Australian clinical mastitis studies conducted in the 1960s found that the most common isolates were *Streptococcus agalactiae* and *S. aureus* (4, 5). Depending on the study, *S. agalactiae* was isolated in 70–100% of herds and *S. aureus* in 97–100% of herds (4–6). It is generally accepted that improvements in milking hygiene have reduced mastitis caused by contagious pathogens such as *S. agalactiae*. However, the relative incidence of environmental pathogens such as *S. uberis* has increased (3, 7, 8).

Australia has approximately 5,700 dairy farms across eight dairy regions, with most milk production occurring in southeast Australia [Murray Dairy (22%), Western Victoria (22%), and the Gippsland Region, Victoria (21%)] (9). In Australia, there has been an intensification of the dairy industry with an overall decrease in the number of farms; however, herd size per farm has increased (10, 11). The majority of dairy farms are pasture-based, with 60–65% of the diet of dairy animals made up of grazed pasture and the remainder supplementary feeds (grains, silage, and hay) (11). This does vary with region, and partial mixed rations and the hybrid system are used most commonly in Queensland (23%), New South Wales (NSW) (22%), Murray Dairy (21%), and Western Australia (WA) (20%) (11). Total mixed rations account only for 1% of farms. The most common milking shed designs use herringbone and rotary milking machines, with a small number of automatic milking systems in use across Australia (12). Calving patterns also vary between regions, with year-round calving most common in Queensland, NSW, South Australia (SA), and WA, while farms in Victoria use seasonal, year-round, or split calving, and in Tasmania, it is primarily seasonal calving (11).

These regions vary in climate and farm practices, which in turn may affect the etiology of clinical mastitis (7). In WA between April and September 2020, *Bacillus* spp., coagulase-negative staphylococci (CoNS), and *Pseudomonas* spp. were the most common bacteria isolated from cases of clinical mastitis (13). However, in southeast Australia from 2011 to 2012, *S. uberis* and *S. aureus* were the most common pathogens cultured from clinical mastitis samples (3). Understanding the pathogenic causes of clinical mastitis in different dairy regions helps in designing targeted mastitis control and prevention strategies.

Treatment of mastitis is the major reason for antimicrobial use in dairy cattle in Australia. Other reasons include lameness, gastrointestinal and reproductive disease, and, to a lesser extent, respiratory disease. Various antimicrobials are used to treat mastitis (e.g., penethamate, penicillin, ampicillin, oxytetracycline, oxytetracycline/oleandomycin/neomycin, tylolsin, amoxicinllinc/clavualnaic acid, cloxacillin, cloxacillin/ampicillin, cloxacillin/penicillin, cephalonium, cephapirin, cefuroxime, and trimethoprim/sulfamethoxazole) with cloxacillin identified as the first choice by veterinarians to treat clinical mastitis and use in dry cow therapy (14, 15). Treatment of mastitis increases the risk of antimicrobial residues in milk and has the potential to lead to the development of antimicrobial resistance (16). Surveillance and monitoring are important parts of the response to antimicrobial resistance (17). Research indicates that, in Australia, common mastitis-causing bacteria have generally remained susceptible to the antimicrobials used in their treatment. Low levels of resistance to penicillin, amoxicillin, and erythromycin in *S. aureus* isolates have been reported in the cases of bovine mastitis (3, 18). A study of 203 *S. aureus* mastitis isolates detected no methicillin resistance (19); however, a recent study has identified 25% (*n* = 9) of their mastitis samples (clinical and sub-clinical combined) as methicillin-resistant *S. aureus* (13). In a recently published study of mastitis in southeast Australian dairy herds, *S. uberis* isolates were susceptible to amoxicillin, cloxacillin, and penicillin; however, resistance was identified at low levels against erythromycin and tetracycline (3). In the same study, *E. coli* isolates demonstrated moderate resistance to streptomycin and neomycin (3).

The aims of this study were to (1) describe the mastitis-causing organisms isolated from bovine milk samples submitted to four veterinary diagnostic laboratories in Australia between 2015 and 2019, (2) determine any geographical, temporal, and seasonal patterns for the most common pathogens, and (3) summarize the antimicrobial susceptibility data for the most commonly isolated pathogens.

## 2. Methods

### 2.1. Case selection

**Figure 1:**
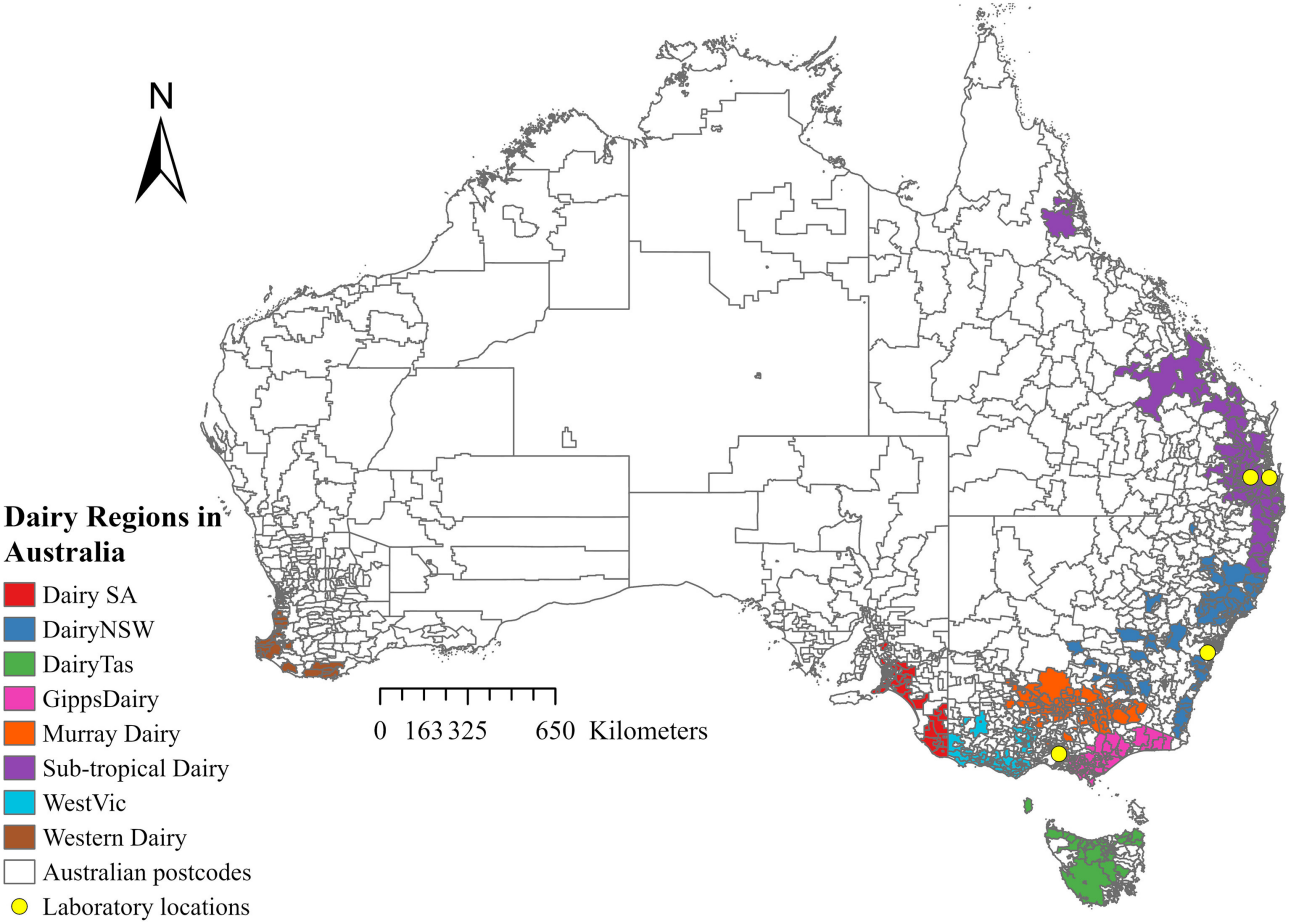
Map showing the eight Australian dairy regions and the location of each laboratory.

Records from four Australian veterinary diagnostic laboratories were obtained for all milk samples submitted between 1 January 2015 and 31 December 2019. The laboratories were Gribbles Veterinary Pathology Clayton, Victoria; Biosecurity Sciences Laboratory, Coopers Plains, Queensland; The University of Queensland, School of Veterinary Science Veterinary Laboratory Service, Gatton, Lawes, Queensland; and Queensland and Elizabeth Macarthur Agricultural Institute Veterinary Laboratory, Menangle, New South Wales (Figure 1). Information collected from the records included bacterial culture and antimicrobial susceptibility results, date of submission, and postcode, when available. Clinical information and details of sample collection were often inconsistent or missing. No data were recorded on whether the sample came from clinical or subclinical mastitis or whether the milk was from individual cow quarters, composite milk, pooled samples, or bulk tank milk samples.

### 2.2. Microbiological methods

Microbiological methods within each laboratory were examined, and all laboratories cultured milk samples using standard veterinary diagnostic techniques (20, 21). Briefly, between 10 and 100 μl of milk was inoculated onto Columbia Sheep Blood Agar (SBA) at all laboratories, and Gribbles Veterinary Pathology, Biosecurity Sciences Laboratory, and Veterinary Laboratory Service also inoculated Edwards medium and MacConkey agar (MCA). Plates were incubated aerobically (with/without 5% CO_2_) at 37°C and examined several times between 18 and 72 h of incubation, depending on the laboratory. The laboratory at the Elizabeth Macarthur Agricultural Institute performed an additional incubation of the original milk sample for approximately 18 h at 37°C and streak-plated to obtain single colonies if insufficient or no growth occurred on the initial culture plate. In all laboratories, colonies were sub-cultured onto SBA and incubated at 37°C overnight to achieve pure cultures. Colonies were identified by colony morphology, gram stain, and biochemical and serological testing, including catalase, oxidase, Microbact-24E or−12S (Oxoid), API^®^ RAPID ID 32 E (bioMérieux), Streptococcus Lancefield grouping (Oxoid), cystine tryptic agar, sugar fermentation (glucose, maltose, lactose, and sucrose), and urease activity. After mid-2017 and from 2019, Biosecurity Sciences Laboratory and Elizabeth Macarthur Agricultural Institute, respectively, identified bacterial isolates using a Bruker MALDI Biotyper^®^. A pure growth of a known mastitis-causing pathogen or a known pathogen isolated in lightly mixed growth as the predominant organism was considered significant. For example, *S. agalactiae* or *S. aureus* in a mixed growth was considered significant. A culture of three or more organisms was considered mixed and not significant (20).

### 2.3. Data management

For analysis, bacteria were reported at species level if more than 100 isolates were cultured and at genus level if the number of isolates was between 25 and 100. Coagulase-negative staphylococci (CoNS) were grouped. Bacterial isolates cultured at a low level (< 25 isolates) were grouped and reported as other bacteria. Fungi and yeast were reported as eukaryotic organisms.

Sample locations were categorized by “dairy region” for all samples with valid postcode data. This category was based on the Dairy Australia farming regions, as shown in Figure 1. Dairy region postcode data was supplied by Dairy Australia (Dairy Australia, personal communication, 10 August 2022). The postcode of each farm was allocated into one of seven regions, which included Dairy South Australia (DairySA), Dairy New South Wales (Dairy NSW), Dairy Tasmania (DairyTas), Gippsland Dairy (GippsDairy), Murray Dairy, Sub-tropical Dairy, and Western Victoria Dairy (WestVic Dairy). No samples were submitted from the Western Dairy region.

### 2.4. Statistical analysis

Statistical analysis was performed using STATA 14.1 and 17 (Stata Corporation, College Station, Texas, United States). The number and percentage of each culture result were calculated overall with a 95% binomial exact confidence interval for the years 2015–2019 and by dairy region. The number and percentage of samples that may not require antibacterial treatment (gram-negative, eukaryotic organisms, and no growth) were calculated.

The unconditional association between the seven most isolated mastitis-causing organisms and the risk factors (year, season, and dairy region) was evaluated by conducting a univariate logistic regression analysis. Due to the low number of samples submitted from Dairy Tasmania, this dairy region was not included in the analysis. The analysis was conducted separately for each pathogen. Risk factors with a *P*-value of ≤ 0.15 were included in the multivariable logistic regression (22).

Multivariable logistic regression models were built using a backward stepwise elimination procedure. At each step, the variable with the highest *P*-value was removed until all variables retained in the final model had *P*-values of < 0.05. The Hosmer-Lemeshow goodness-of-fit statistic was used to assess the fit of the final model. In addition, the area under the receiver operating characteristic (ROC) curve was estimated to assess the predictive power of the model (23).

The one-way random effect models were employed to assess the correlation between pathogen occurrence and the postcode of dairy farms (24) by estimating the individual intraclass correlation coefficient (ICC):


$$\begin{array}{l} \rho =ICC=Corr\left(y_{ij\;},y_{ij^{{}^{\prime }}}\right)=\frac{\sigma_{r}^{2}}{\sigma_{r}^{2}+\;\sigma_{\epsilon }^{2}}, \end{array}$$

where $\sigma_{r}^{2}\;$refers to the variance between postcodes of dairy farms and $\sigma_{\epsilon }^{2}\;$refers to error variance or variance within postcodes of dairy farms. The ICC was estimated for *S. aureus, E. coli, S. uberis, S. dysgalactiae, S. agalactiae, C. bovis*, and *Nocardia* spp.

In the ICC random-effects models, the number of positive and negative samples for a particular pathogen was considered “raters” to their occurrence status in a specific postcode of dairy farms (represented as “targets”):


$$\begin{array}{l} y_{ij}=\mu +r_{i}+\;\epsilon_{ij}, \end{array}$$

where μ is the mean rating; *r*_*i*_ is the target random effect; ϵ_*ij*_ is the random error; and *y*_*ij*_ is the *j*^th^ rating on the *i*^th^ target (*I* = 1,…, *n* and *j* = 1,…, *k*) (25). The significance of the estimated ICC was evaluated using the F-test (25).

For the pathogens with evidence of significant clustering based on an ICC at the postcode level of a *p*-value of < 0.05, the proportions of those pathogens were calculated as the number of cultured isolates divided by the total number of cultured isolates. To visualize areas with a high proportion of the cultured pathogens, the results were mapped as choropleth maps per postcode for the entire study period in ArcGIS Pro version 2.5.0 (Esri, Redlands, CA, United States) using the proportions data and the postal areas polygon map extracted from the Australian Statistical Geography Standard (ASGS) Edition 2016 digital boundaries (26) in the format of an ESRI Shapefile. The dairy regions were visualized using extent maps for both bacteria.

Spatial clustering analysis using global (27) and local Moran's I in the form of Local Indicators of Spatial Association (LISA) (28) was performed using the proportions of pathogens with an ICC of a *p-*value of < 0.05 for the entire study period. Visualizing the proportional data as choropleth maps informed us as to which areas should be accounted for in the spatial clustering analysis. To account for the connections between all dairy regions at postcode level, the postcodes within the dairy regions and surrounding areas were extracted from ArcGIS Pro version 2.5.0 (Esri, Redlands, CA, United States) along with the proportions data as a GeoPackage. Null values were replaced by zeros. The GeoPackage was imported into GeoDa version 1.18.0, where the queen contiguity was used to calculate global Moran's I and LISA cluster analysis using a Monte Carlo simulation of 999 permutations and a *P*-value of 0.05 for significance (29). This generated a global Moran's I index value, pseudo *P*-value, and Z-score for both bacteria. LISA cluster analysis categorized postcodes as significant spatial clusters, including hotspots (High-High) and coldspots (Low-Low), as spatial outliers (High-Low and Low-High), and as not significant postcodes. The LISA cluster analysis results were extracted from GeoDa and visualized as LISA cluster maps in the ArcGIS Pro version 2.5.0 (Esri, Redlands, CA, United States) for *S*. *aureus* and *S. uberis*.

### 2.5. Antimicrobial susceptibility testing

Antimicrobial susceptibility data from cultured bacteria for submitted samples were collected from three laboratories; it was not available from the Elizabeth Macarthur Agricultural Institute. Disc diffusion antimicrobial susceptibility testing was performed following the Veterinary Clinical and Laboratory Standard Institute (CLSI) guidelines (30–33). *S. aureus* ATCC 25923, *E. coli* ATCC 25922, and *S. pneumoniae* ATCC 49619 were included as quality control (30–33).The number and percent of susceptible isolates were calculated for *S. aureus, S. uberis, S. dysgalactiae, S. agalactiae*, and coliform bacteria for a range of antimicrobials.

## 3. Results

### 3.1. Descriptive summary of laboratory data

When microbiological culture results from all laboratories were combined, the dataset consisted of records from 22,102 milk samples submitted between 2015 and 2019. Most of the sample results were from Gribbles (*n* = 19,547). Approximately 83% (*n* = 16,293) of the samples submitted to this laboratory came from Victoria, 4.6% (*n* = 905) from South Australia, 2.8% (*n* = 543) from New South Wales, 1% (*n* = 207) from Queensland, and 0.05% (*n* = 10) from Tasmania; 8% (*n* = 1,589) of samples had no state recorded. From the Elizabeth Macarthur Agricultural Institute laboratory, 1,546 sample results were collected, most of which were submitted from New South Wales (*n* = 1,430) and a small number from Victoria (*n* = 5), and 111 samples had no state recorded. Both the Veterinary Laboratory Service and Biosecurity Sciences Laboratory only had samples submitted from Queensland, with 209 and 800 samples, respectively. Generally, there was a trend of decreasing sample submission over the course of the study period (5,695 in 2015 to 1,874 in 2019), except for 2017 (*n* = 6,562), which had the highest submission rate over the study period (Table 1). There was a slight increase in the no growth results over time, with the lowest proportion of no growth results recorded in 2015 at 23.6%, with an increase to 30.5% in 2018 and 29.1% in 2019 (Table 1). The number of mixed/contaminated results decreased over time, with the highest in 2015 at 26.2% and the lowest in 2018 at 16.7%. The majority of isolates maintained a consistent proportion over time (Table 1).

**Table 1:** Number and percent of bacterial culture and isolate results from milk samples submitted to four veterinary diagnostic laboratories in Australia from 2015 to 2019, stratified by year.

	**2015**	**2016**	**2017**	**2018**	**2019**	**All years**	
**Isolate**	***n*** **(%)**	***n*** **(%)**	***n*** **(%)**	***n*** **(%)**	***n*** **(%)**	***n***	**Total percent (95% CI)**
**Culture result**							
Samples submitted	5,695 (100.0)	4,162 (100.0)	6,562 (100.0)	3,809 (100.0)	1,874 (100.0)	22,102	100.0
No growth	1,344 (23.6)	994 (23.9)	1,738 (26.5)	1,161 (30.5)	545 (29.1)	5,782	26.2 (25.6–27.0)
Mixed/contaminated	1,492 (26.2)	999 (24.)	1,409 (21.5)	635 (16.7)	378 (20.2)	4,913	22.2 (21.7–22.8)
Positive growth	2,859 (50.2)	2,169 (52.1)	3,415 (52.0)	2,013 (52.8)	951 (50.7)	11,407	51.6 (51.0–52.3)
**Isolate result**							
Total isolates	2,977 (100.0)	2,267 (100.0)	3,579 (100.0)	2,078 (100.0)	1,006 (100.0)	11,907	100.0
*Streptococcus uberis*	1,220 (41.0)	815 36.0)	1,475 (41.2)	1,002 (48.2)	400 (39.8)	4,912	41.3 (40.4–42.1)
*Staphylococcus aureus*	699 (23.5)	537 (23.7)	803 (22.4)	521 (25.1)	246 (24.5)	2,806	23.6 (22.8–24.3)
*Escherichia coli*	265 (8.9)	178 (7.9)	255 (7.1)	136 (6.5)	118 (11.7)	952	8.0 (7.5–8.5)
*Streptococcus dysgalactiae*	130 (4.4)	202 (8.9)	304 (8.5)	84 (4.0)	62 (6.2)	782	6.6 (6.1–7.0)
*Corynebacterium bovis*	237 (8.0)	92 (4.1)	204 (5.7)	99 (4.8)	46 (4.6)	678	5.7 (5.3–6.1)
*Streptococcus agalactiae*	61 (2.0)	28 (1.2)	170 (4.7)	64 (3.1)	10 (1.0)	333	2.8 (2.5–3.1)
*Nocardia* spp.	53 (1.8)	97 (4.3)	89 (2.5)	42 (2.0)	33 (3.3)	314	2.6 (2.4–2.9)
Other *Streptococcus* spp.	39 (1.3)	67 (3.0)	27 (0.8)	9 (0.4)	5 (0.5)	147	1.2 (1.0–1.4)
CoN^a^ *Staphylococcus* spp.	42 (1.4)	43 (1.9)	26 (0.7)	6 (0.3)	6 (0.6)	123	1.0 (0.9–1.2)
*Serratia* spp.	20 (0.7)	18 (0.8)	48 (1.3)	12 (0.6)	19 (1.9)	117	1.0 (0.8–1.2)
*Trueperella* spp.	33 (1.1)	30 (1.3)	29 (0.8)	12 (0.6)	5 (0.5)	109	0.9 (0.8–1.1)
Eukaryotic organism	40 (1.3)	20 (0.9)	31 (0.9)	12 (0.6)	5 (0.5)	108	0.9 (0.7–1.1)
*Pasteurella multocida*	28 (0.9)	24 (1.1)	28 (0.8)	15 (0.7)	8 (0.8)	103	0.9 (0.7–1.0)
*Bacillus* spp.	26 (0.9)	20 (0.9)	15 (0.4)	23 (1.1)	5 (0.5)	89	0.7 (0.6–0.9)
*Klebsiella* spp.	26 (0.9)	24 (1.1)	13 (0.4)	13 (0.6)	5 (0.5)	81	0.7 (0.5–0.8)
*Pseudomonas* spp.	17 (0.6)	17 (0.7)	18 (0.5)	9 (0.4)	11 (1.1)	72	0.6 (0.5–0.8)
Other bacteria	41 (1.4)	55 (2.4)	44 (1.2)	19 (0.9)	22 (2.2)	181	1.5 (1.3–1.8)

^a^CoN, coagulase negative. The culture result is shown as the number and percentage of samples submitted. The isolate results are shown as the number and percentage of total isolates cultured.

The isolates were re-classified into 17 categories, *Corynebacterium bovis, Escherichia coli, Pasteurella multocida, S. aureus, S. agalactiae, S. dysgalactiae*, and *S. uberis* were all reported at the species level. Coagulase-negative staphylococci (CoNS) included *S. chromogenes, S. epidermidis, S. haemolyticus, S. hyicus, S. sciuri, S. simulans, S. warneri, S. xylosus, Staphylococcus* spp., and coagulase-negative *Staphylococci*. Other isolates were grouped as follows: *Bacillus* spp. included *B. cereus, B. licheniformis, B. thuringiensis*, and *Bacillus* sp.; *Klebsiella* spp. included *K. oxytoca, K. pneumoniae*, and *Klebsiella* sp.; *Nocardia* spp. included *N. asteroides* and *Nocardia* spp.; other streptococci included *S. bovis, S. equinus, S. gallolyticus, Streptococcus* group D (which may include non-enterococcal and enterococcal), *S. lutetiensis, S. parauberis, S. pneumoniae, S. suis, S. viridans*, and *Streptococcus* sp.; *Pseudomonas* spp. included *P. aeruginosa, P. flavescens, P. fluorescens*, and *Pseudomonas* sp.; *Serratia* spp. included *S. liquefaciens, S. marcescens*, and *Serratia* sp.; *Trueperella* spp. included *Trueperella pyogenes* and *Actinomyces/Trueperella* sp.; and other bacteria included *Acinetobacter baumannii, Acinetobacter* spp., *Aerococcus viridans, Aeromonas hydrophila, Aeromonas* sp., *Bibersteinia trehalosi, Burkholderia cepacia, Citrobacter freundii, C. koseri, Citrobacter* sp., *Corynebacterium-*like organism, *Corynebacterium* sp., *Cronobacter sakazakii, Enterobacter aerogenes, Enterobacter cloacae, Enterobacter* sp., *Enterococcus durans, Enterococcus faecalis, Enterococcus faecium*, gram-negative bacilli, gram-positive bacilli, gram-positive branching filaments resembling *Actinomyces* sp., gram-positive cocci, *Helcococcus ovis, Histophilus somni, Histophilus* sp., *Lactococcus garvieae, Lactococcus* sp., *Lactococcus lactis, Lelliottia aminigena, Leuconostoc* spp., *Listeria monocytogenes, Mannheimia haemolytica, Mannheimia varigena, Micrococcus* spp., *Mycobacterium* sp., *Mycoplasma* sp., *Pantoea* sp., *Pantoea agglomerans, Proteus mirabilis, Proteus* sp., *Raoultella ornithinolytica, Salmonella* spp., *Shewanella* sp., *Staphylococcus/Micrococcus* spp., *Vibrio alginolyticus, Yersinia enterocolitica, Y. pseudotuberculosis*, and *Y. ruckeri*. Eukaryotic organisms included *C. krusei, C. lusitaniae, C. tropicalis*, and *Candida* spp., Fungus, *Prototheca* sp., *Scedosporium prolificans, Trichosporon* sp. and yeast.

**Figure 2:**
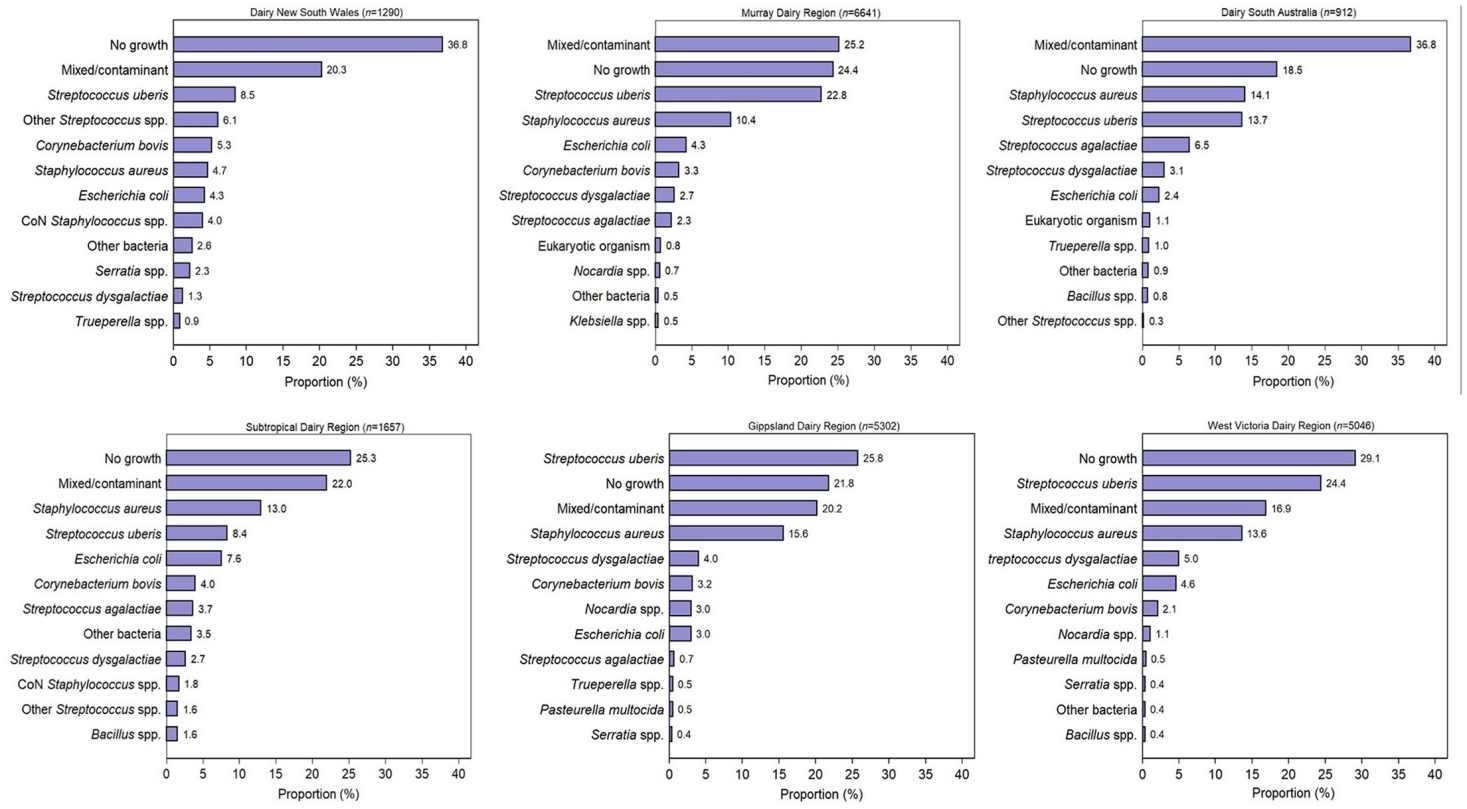
The most common 12 bacterial culture results by Dairy Australia region for milk samples submitted to four veterinary diagnostic laboratories in Australia from 2015 to 2019. CoN, coagulase negative.

Culture results were reported as positive growth for at least one significant pathogen (*n* = 11,407; 51.6%), no growth (*n* = 5,782; 26.2%), and mixed/contaminated growth (*n* = 4,913; 22.2%). A total of 11,907 isolates were reported from 11,407 milk samples with positive growth. Overall, the most common pathogen isolated was *S. uberis* (41.3%), followed by *S. aureus* (23.6%), *E. coli* (8.0%), *S. dysgalactiae* (6.6%), and *C. bovis* (5.7%) (Table 1). In total, 20,880 submitted samples had postcode data available and were able to be assigned to the Dairy Australia regions. Overall, 32% of the samples originated from the Murray Dairy; 25% from the GippsDairy; 24% from the WestVic Dairy, and 8, 6, and 4% from the Sub-tropical Dairy, Dairy NSW, and DairySA regions, respectively. The most common organisms isolated varied by region (Figure 2). *S. uberis* was the most common pathogen identified in the Dairy NSW, Murray Dairy, GippsDairy, and WestVic regions. Whereas, in the Sub-tropical and DairySA regions, *S. aureus* was the most common mastitis pathogen cultured. Consistently, *S. uberis* and *S. aureus* were the two most common pathogens in each region, except for Dairy NSW, where *S. uberis* was the most common, followed by other *Streptococcus* spp., *C. bovis*, and then *S. aureus*. For all regions, no growth and mixed/contaminated results were consistently part of the three most common culture results.

Data for no growth, gram-negative bacteria, and eukaryotic organisms were combined for each region and accounted for 46.4% of submissions from Dairy NSW, 38.4% from Sub-tropical Dairy, 35.2% of WestVic Dairy regions, 31.2% from Murray Dairy, 27.2% from GippsDairy, and 23.4% from DairySA (Table 2).

**Table 2:** Number and percent of bacterial culture results for no growth, gram-negative bacteria, fungi, and yeast for each Dairy Australia region for milk samples submitted to four veterinary diagnostic laboratories in Australia from 2015 to 2019.

	**Dairy region**											
**Culture result**	**Dairy NSW**		**DairySA**		**GippsDairy**		**Murray Dairy**		**Sub-tropical Dairy**		**WestVic Dairy**	
	***n***	**%**	***n***	**%**	***n***	**%**	***n***	**%**	***n***	**%**	***n***	**%**
Total samples submitted	1,290		912		5,302		6,641		1,657		5,046	
No growth	475	37.0	169	19	1,157	22.0	1,621	24.4	419	25.0	1,467	29.0
Gram-negative	116	9.0	30	3.3	244	5.0	399	6.0	210	13.0	309	6.0
Eukaryotic organisms	5	0.4	10	1.1	10	0.2	53	0.8	7	0.4	11	0.2
Total	596	46.4	209	23.4	1,411	27.2	2,073	31.2	636	38.4	1,787	35.2

DairyNSW, Dairy New South Wales; Dairy SA, Dairy South Australia; GippsDairy, Gippsland Dairy; WestVIC, West Victoria Dairy.

### 3.2. Logistic regression

Univariate logistic regression was performed for season, dairy region, and year of sample submission for the seven most common mastitis-causing organisms (Supplementary Tables 1–7). For *S. uberis* and *S. aureus*, there was an association between a positive culture result and the dairy region. All regions except the Sub-tropical Dairy region were more likely to culture *S. uberis* compared to the reference, Dairy NSW (*P* < 0.001). Similarly, for *S. aureus*, a positive culture result was more likely in all other dairy regions compared to Dairy NSW (*P* < 0.001). Multivariable logistic regression was performed; however, none of the models had an acceptable goodness-of-fit test statistic.

### 3.3. Intraclass correlation, visualization, and spatial clustering analysis

The intraclass correlation analysis identified significant clustering for *S. aureus* and *S. uberis* at a *p*-value of < 0.01 (Supplementary Table 8). No clustering was identified for *E*. *coli, S. dysgalactiae, S. agalactiae, Corynebacterium bovis*, and *Nocardia* spp. using intraclass correlation.

**Figure 3:**
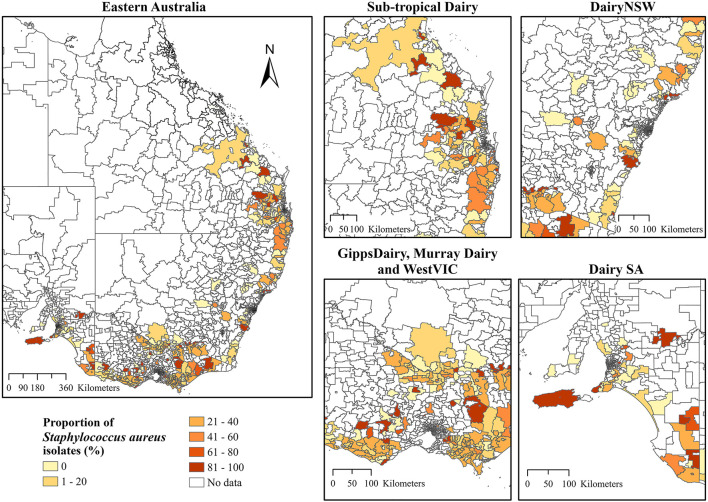
The proportion of *Staphylococcus aureus* isolates (%) cultured from the different Dairy regions at postcode level in Australia. The extent maps display each dairy region. DairyNSW, Dairy New South Wales; GippsDairy, Gippsland Dairy; WestVIC, West Victoria Dairy; Dairy SA, Dairy South Australia.

**Figure 4:**
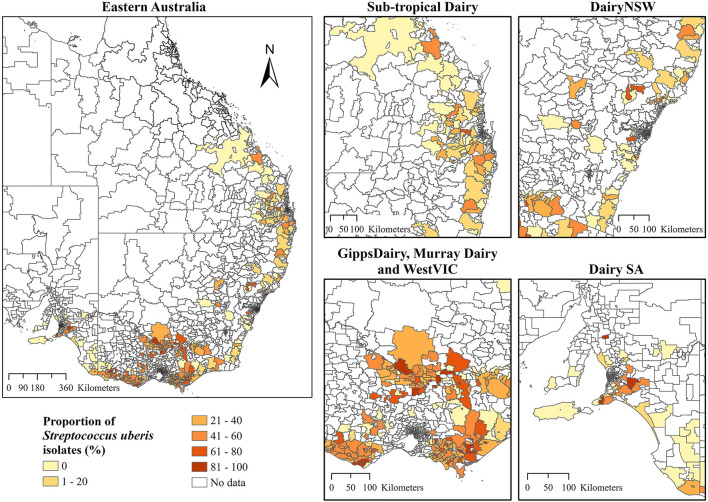
The proportion of *Streptococcus uberis* isolates (%) cultured from the different Dairy regions at postcode-level in Australia. The extent maps display each dairy region. Dairy NSW, Dairy New South Wales; GippsDairy, Gippsland Dairy; WestVIC, West Victoria Dairy; Dairy SA, Dairy South Australia.

Choropleth maps for the proportion of *S. aureus* and *S. uberis* by dairy region indicated a high proportion of *S*. *aureus* isolates that were cultured in Sub-tropical Dairy, WestVic, and DairySA (Figure 3), whereas for *S*. *uberis*, there was a higher proportion cultured in GippsDairy, Murray Dairy, and WestVic postcodes (Figure 4).

**Figure 5:**
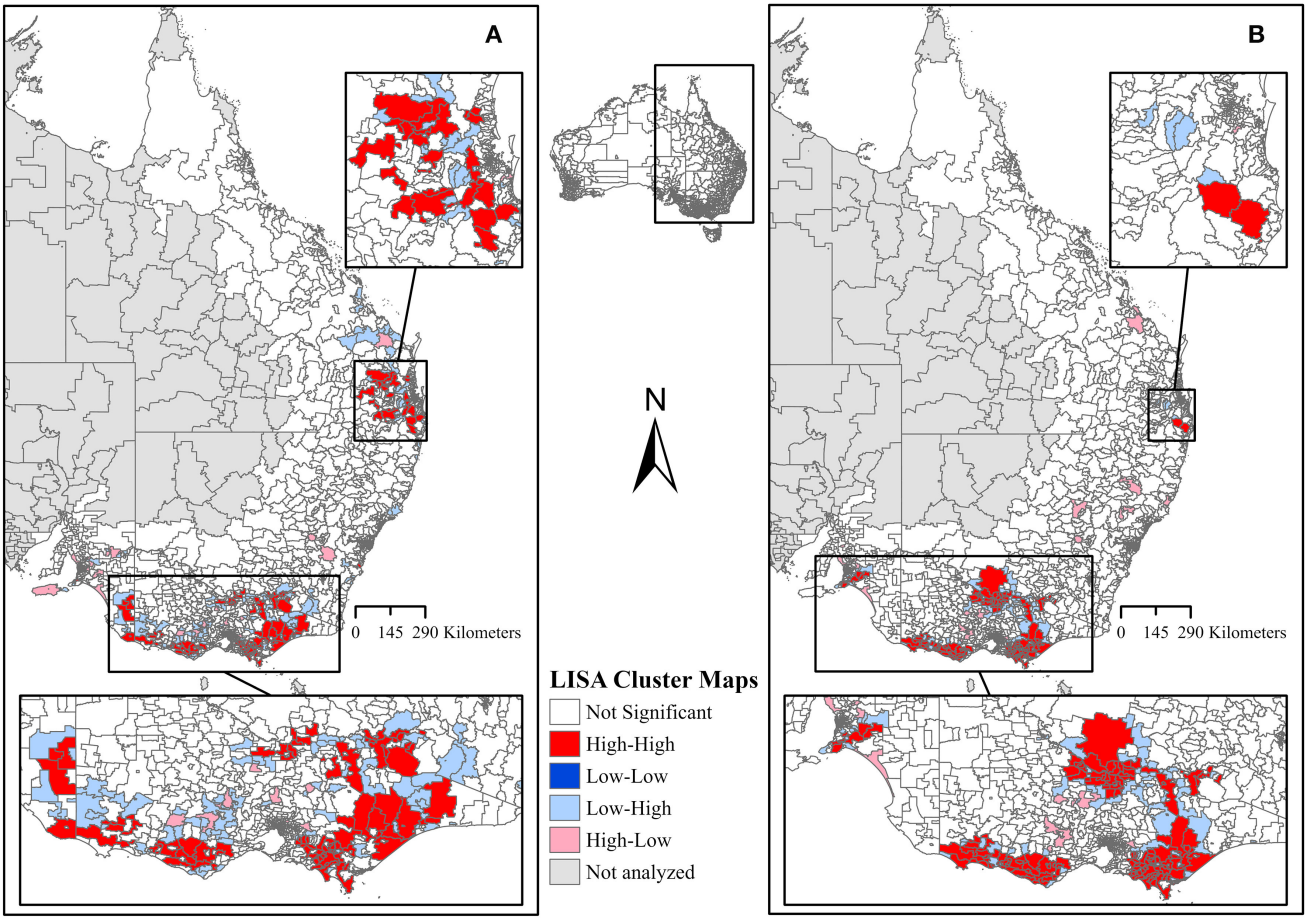
Local Indicators of Spatial Association (LISA) cluster maps of the proportion of **(A)** *Staphylococcus aureus* and **(B)** *Streptococcus uberis* isolates. All dairy regions in this study were analyzed together.

In addition to ICC, the global Moran's I further indicated positive and significant spatial clustering for *S. aureus* and *S. uberis*, necessitating the need to perform LISA cluster analysis (Table 3). The LISA cluster analysis identified 83 High-High (hotspot) postcodes for *S. aureus* and 123 High-High postcodes for *S*. *uberis* across all of the analyzed dairy regions (Table 3). There were no Low-Low (coldspot) postcodes identified for each bacterium (Table 3). Figures 5A, B demonstrate the variability of the hotspots for the proportion of cultured *S. aureus* and *S. uberis* isolates. Eighty-three total hotspot postcodes for *S. aureus* were located within GippsDairy (*n* = 32, High-High postcodes), Sub-tropical Dairy (*n* = 16), Murray Dairy (*n* = 15), WestVic Dairy (*n* = 14), Dairy SA (*n* = 5), and Dairy NSW (*n* = 1), whereas for *S. uberis*, the 123 hotspot postcodes were located in GippsDairy (*n* = 44, High-High postcodes), Murray Dairy (*n* = 38), WestVic (*n* = 32), Dairy SA (*n* = 7), Sub-tropical Dairy (*n* = 2), and none were found in Dairy NSW (Figures 5A, B).

**Table 3:** Global Moran's I spatial autocorrelation and the number of clustered postcodes identified through the Local Indicators of Spatial Association (LISA) analysis for the proportion of *Staphylococcus aureus* and *Streptococcus uberis* isolates, 2015–2019.

	**Isolates**	
**Spatial clustering analysis results**	***Staphylococcus aureus***	***Streptococcus uberis***
**Global Moran's I**		
Moran's I index	0.1397	0.4137
Z-score	9.4318	29.0148
*p*-value	0.001	0.001
**Number of postcodes in LISA cluster analysis (*****N** =* **2,021)**		
High-High (hotspots)	83	123
Low-Low (coldspots)	0	0
Low-High	136	76
High-Low	23	20
Not significant	1,779	1,802

### 3.4. Antimicrobial susceptibility

*Staphylococcus aureus* had a high susceptibility to beta-lactam antimicrobials, with susceptibilities of 87.7–100% (Table 4). There was some resistance to erythromycin, with 83.3% of *S. aureus* being susceptible. *S. uberis* was highly susceptible to all beta-lactam antimicrobials (99.9–100%), tetracycline (99.4%), and trimethoprim-sulfamethoxazole (95.5%). However, only 75.9% of *S. uberis* isolates were susceptible to erythromycin and 84.6% to lincosamides. Resistance against tetracycline was found among *S. dysgalactiae* isolates, with only 20.1% being susceptible; however, high susceptibility was recorded for all beta-lactam antimicrobials (99.7–100%). *S. agalactiae* is highly susceptible to beta-lactams (99.3–100%), tetracycline (97.1%), and trimethoprim-sulfamethoxazole (100%). Coliforms showed high susceptibility to amoxicillin-clavulanic acid (92.4%), second-generation cephalosporins (97.0%), third-generation cephalosporins (96.5%), and trimethoprim-sulfamethoxazole (91.5%). They had lower susceptibility to amoxicillin/ampicillin (67.6%), neomycin (78.3%), and tetracycline (88.8%).

**Table 4:** Number of tests for antimicrobial susceptibility and percentage susceptible (S%) of five mastitis-causing bacterial pathogens from three commercial veterinary diagnostic laboratories in Australia over a 5-year period (2015–2019).

	***Staphylococcus aureus*** **(*****n** **=*** **2,675)**		***Streptococcus uberis*** **(*****n** **=*** **4,785)**		***Streptococcus dysgalactiae*** **(*****n =*** **764)**		***Streptococcus. agalactiae*** **(*****n** **=*** **328)**		**Coliforms (*****n** **=*** **993)**		
**Antimicrobials**	**No. tests**	**S %**	**No. tests**	**S %**	**No. tests**	**S %**	**No. tests**	**S %**	**No. tests**	**S %**	**Total no. tests**
Cloxacillin	2.,497	99.9	4,480	99.9	702	100	262	100	9	44.4	7,950
Penicillin	2,445	87.8	4,494	99.9	704	100	261	99.6	11	0	7,915
Ampicillin/amoxicillin	2,500	87.7	4,521	99.9	722	99.7	274	99.3	882	67.6	8,899
Amoxicillin/clavulanic acid	63	100	55	100	21	100	12	100	877	92.4	1,028
First-generation cephalosporin	3	100	14	100	2	100	0	-	24	50	43
Second-generation cephalosporin^a^	2,441	99.9	4,476	100	702	100	261	100	796	97	8,676
Third-generation cephalosporin^b^	2	100	5	100	1	100	0	-	37	96.5	45
Penicillin/novobiocin	2,450	99.9	4,488	100	707	100	262	100	20	5	7,927
Erythromycin	60	83.3	54	75.9	22	90.9	13	100	15	6.7	164
Lincosamide	1	100	13	84.6	2	50	0	-	0	-	16
Neomycin^c^	58	96.5	41	14.6	20	10	13	15.4	60	78.3	192
Tetracycline^d^	2,503	99.7	4,535	99.4	723	20.1	274	97.1	884	88.8	8,919
Trimethoprim-sulfamethoxazole	2,498	100	67	95.5	23	91.3	13	100	883	91.5	3,484

^a^Cefuroxime. ^b^Ceftazidime, ceftiofur (34). ^c^Streptococci are reported to be intrinsically resistant to aminoglycosides, neomycin (35). ^d^Tetracycline is used to predict the efficacy of oxytetracycline and chlortetracycline (36).

## 4. Discussion

Microbiological culture results from four Australian veterinary diagnostic laboratories identified that at least one significant organism was isolated from 51.6% of samples, with the remainder of samples either having no growth (26.1%) or mixed/contaminated growth (22.2%). Sample submission over the course of the study generally declined, with the lowest number of samples submitted in 2019; however, there was a peak in sample submission in 2017. Climatic factors such as floods and droughts may have affected the submission pattern. In early 2017, tropical cyclone Debbie caused large rainfall events along the east coast of Queensland and northern New South Wales, which led to flooding in several areas (37) including the dairy regions in our study. As mastitis is more common after rain, these events could, at least in part, have increased the incidence of mastitis and thus of culture submissions in 2017. In contrast, 2019 was the driest year on record for many parts of Australia (38); thus, dry conditions may have led to a decrease in mastitis. In addition, the on-going drought increased dairy farm costs due to the need to supplement feed and water (39); therefore, the extra cost associated with bacterial culture may not have been feasible for producers during this time. Future research should investigate how climatic events affect mastitis in Australia, especially in relation to environmental pathogens such as *S. uberis* and *E. coli*.

No growth and mixed/contaminated results accounted for approximately half (49%) of the overall culture results. Using real-time PCR, Taponen et al. (40) found that a substantial proportion (43%, *n* = 79) of no-growth samples were positive for at least one of 11 common mastitis-causing bacteria. No growth results from bacterial culture may be attributed to poor bacterial viability in the sample due to the antibacterial factors of milk (40), the presence of bacteria that cannot be cultured in standard media or under the conditions offered, or the infection already being cleared at the time of sampling, as often occurs with gram-negative infections (41). Mixed/contaminated samples indicate a failure to collect a sterile sample and highlight the difficulties farmers have in collecting milk samples in the dairy environment. No growth and mixed/contaminated culture results are frustrating for the farmer, and the high rate of negative results is known to be a factor limiting sample submissions. On-going education is required to emphasize the importance of a clean sample to reduce the number of contaminated culture results.

No growth results combined with gram-negative bacteria and eukaryotic organisms represent a subset of mastitis cases for which antibiotic treatment may not be warranted. These results combined accounted for between 23 and 46% of the samples submitted for each region. Laboratory culture takes a minimum of 48 h to conduct, with Australian farmers reporting results are generally provided 3–10 days after sample submission to their veterinarian. In order to improve antimicrobial stewardship, the results are required quickly, as treatment can often be delayed for 24 h without serious consequences, while longer delays may result in poorer outcomes (42). Recently, several on-farm tests that can provide results in 24 h, such as the Mastatest^®^ and on-farm rapid culture, have been developed. Diagnostic tests that can deliver rapid results offer the opportunity for dairy farmers to move to targeted therapy for clinical mastitis. Targeted therapy may be especially useful in instances of no growth or gram-negative results where antibiotic treatment could be withheld. Not only does this improve antimicrobial stewardship but also economic benefits, such as reduced treatment costs and lost milk revenue. However, it is important when contemplating new diagnostic methods that they should be evaluated against the World Health Organization's ASSURED criteria (accuracy, sensitivity, specificity, user-friendliness, being rapid or robust, equipment-free, and being deliverable) (43).

This study identifies *S. uberis* and *S. aureus* as the most common organisms isolated from bovine milk samples submitted to four diagnostic laboratories. *S. uberis* was the most common pathogen found in 41.3% of instances of organisms cultured. This finding is consistent with research conducted in 2011–2012 on 65 dairy farms with various feed systems in southeast Australia (3). In contrast, a 2020 study on 12 pasture-based farms in Western Australia identified that *S. uberis* only accounted for 2.5% of clinical mastitis isolates, with *Bacillus* spp. (30.5%) being the most common isolate (13). *S. aureus* was the second most common pathogen isolated in our study (23.6%), which was once again consistent with the southeast Australian dairy study (3). These results differ from older research, which found *S. aureus* comprised 58.2% of total isolates from cases of mastitis in Queensland (44). In the same study, *S. agalactiae* was the second-most isolated pathogen at 19%, which was much greater than the 2.8% recorded in our study. Over time, a decrease in the isolation of contagious mastitis pathogens, such as *S. aureus* and *S. agalactiae*, has been reported worldwide (8, 45). The decrease in contagious pathogens is commonly attributed to the implementation of improved milking hygiene strategies such as milkers wearing gloves and disinfecting teats post-milking (8). Our research supports the idea that contagious mastitis pathogens have decreased over time in Australian dairy herds.

Owing to the limited use of MALDI-TOF during the study period, there is the possibility that some isolates may have been misclassified as *S. uberis* when they were *Lactococcus, Enterococcus*, or *Aerococcus* species (46). Studies from the United States report that *Lactococcus* spp. are commonly isolated from clinical mastitis samples (47) and have been referred to as an emerging clinical mastitis pathogen (48). However, in Australian studies that have used MALDI-TOF for identification, the prevalence of *Lactococcus* spp. remains low (13, 49). Therefore, although it is possible that some bacteria have been misclassified, the number of misclassifications is likely very low.

For *S. uberis* and *S. aureus*, there was an association between isolating an organism and the dairy region, as indicated by the univariate logistic regression. For the Dairy NSW, Murray Dairy, GippsDairy, and WestVic regions, *S. uberis* was the predominant pathogen identified, while for the Sub-tropical and DairySA regions, *S. aureus* was most common. In Australia, common mastitis pathogens are known to vary by region. *S. uberis* has previously been identified as the most predominant mastitis-causing pathogen in Gippsland, Northern Victoria, and Western Victoria (3). Whereas, in Western Australia, *Bacillus* spp. were the most common bacteria isolated from cases of clinical mastitis (13). As the ICC analysis identified overall clustering for *S. aureus* and *S. uberis* at postcode level, it was important to determine the locations of the clustering. Thus, LISA clustering was used to identify hotspot postcodes with a high proportion of cultured *S. aureus* or *S. uberis* for the entire study period for all dairy regions combined. Differences in hotspot postcode locations for the two bacteria may represent true regional differences in pathogen presence; however, there are other confounding risk factors such as herd-level practices (50, 51) and climatic conditions (52, 53) that may more accurately explain these observed variations. Evidence of spatial clustering combined with an understanding of the associated risk factors for individual pathogens could lead to the implementation of more targeted mastitis control protocols based on the hotspot areas.

In this study, the isolates examined showed overall good susceptibility to a range of antimicrobials. When interpreting antimicrobial susceptibility results, it is important to consider the limitations. Although antimicrobial susceptibility plays a role in the treatment of mastitis, *in vitro* susceptibility testing does not necessarily correlate with treatment outcomes (54). For example, in cases of *S. aureus* infection, there are other factors such as parity, days in milk, number of infected quarters, and conformation that can also influence cure (55). In addition, antimicrobial susceptibility clinical breakpoints for bovine mastitis are only available for the intramammary application of ceftiofur, penicillin/novobiocin, pirlimycin, and cefoperazone for some mastitis-causing pathogens (36, 56). Despite these limitations, it is important to conduct antimicrobial susceptibility testing to monitor changes in resistance patterns over time.

Despite overall good susceptibility, resistance to certain isolates in our study was greater than previous research in similar regions. For example, in this study, *S. uberis* isolates were moderately resistant to erythromycin (24%), whereas a previous Australian study found that only 7% of *S. uberis* isolates were resistant to this antimicrobial (3). For *S. aureus*, we found 12% of isolates were resistant to amoxicillin and penicillin and 17% of isolates were resistant to erythromycin compared to 2% for amoxicillin and penicillin and 3% for erythromycin in a previous study (3). Interestingly, in the same study, the risk of *S. aureus* demonstrating resistance to penicillin was 5.2 times higher for subclinical isolates compared to clinical isolates and 4.7 times higher for amoxicillin (3). It is possible that antimicrobial resistance has increased since data collection for Dyson (3) that occurred in 2011 and 2012; however, another plausible explanation for these differences is the methodology used in the two studies. In this study, 80% of *S. dysgalactiae* were resistant to tetracycline, and this is similar to previous studies in Australia (90%) (3), New Zealand (89%) (57), Portugal (90–100%) (58, 59), China (100%) (60), and Canada (82%) (61). Tetracycline resistance has been shown to be associated with tetracycline resistance genes (*tetD, tetM, tetK*, and *tetO*) and mobile genetic elements (58, 60). Resistance is believed to be associated with the long-time use of tetracyclines to treat bovine infections, the ability for resistance to persist in the absence of selection pressure, and its ability to transfer between bacterial genera (61, 62). Tetracycline is not recommended for the treatment of streptococcal mastitis (15), and streptococci remain susceptible to cloxacillin, penicillin, amoxicillin, and cephalosporins. Therefore, despite the long-term use of antimicrobials in the dairy industry, antimicrobial resistance was found to be low for the antimicrobials recommended for use in the treatment of the common mastitis-causing pathogens; however, ongoing monitoring of antibiotic resistance is warranted.

In this study, we have considered that all the samples submitted for bacterial culture were from cattle with either clinical or subclinical mastitis. However, the data in our study were derived from samples that were submitted to laboratories for testing and, therefore, have some limitations. It is important to consider these limitations and acknowledge that the results should only act as a general indication of mastitis in Australia. Several details were not available for the submitted samples. First, details of the case were not disclosed, which meant that it was not possible to differentiate between clinical and subclinical samples. Second, the reason for testing was not reported, meaning that the samples may have been from recurrent cases or cases that failed to respond to treatment, which may have led to an over-representation of certain organisms and an increase in the overall reported level of antimicrobial resistance. Finally, farm details were not available, making it impossible to determine how many samples were submitted from a single farm. Therefore, some farms may be over-represented, potentially biasing results toward organisms found on certain farms rather than regions. Despite these limitations, one of the major strengths of this study was the large number of milk samples analyzed (*n* = 22,102), representing mastitis cases from seven of the eight Australian dairy regions, making this study the largest analysis of mastitis samples conducted in Australia to-date.

## 5. Conclusion

*Streptococcus uberis* and *S. aureus* were the two most common mastitis-causing pathogens isolated from milk samples submitted to four commercial laboratories in Australia. There is an association between dairy region and postcode and the presence of a pathogen; however, further research is required to determine the role of more specific risk factors such as environmental factors and herd-level predictors. A large proportion of milk samples submitted returned a negative culture result (either due to no growth or mixed contaminated growth), and between 23 and 46% of samples from each dairy region may not require antibiotic treatment. This highlights the need for an accurate and reliable on-farm diagnostic test. Overall, there was good antimicrobial susceptibility for the common mastitis-causing pathogens; however, ongoing surveillance is required to facilitate targeted mastitis control and treatment programs.

## Author contributions

Conceptualization: BW, SR, MM, CW, and JG. Methodology: CW, JH, MM, SG, MH, and JG. Data collection: LB, AD, and RP. Data analysis: SG, CW, SH, and CL. Writing—original draft preparation: CL, SG, SH, and JG. Writing—review and editing: CL, SG, SH, CW, BW, LB, AD, RP, MM, MH, SR, JH, and JG. All authors contributed to the article and approved the submitted version.
