# Supplementary material for: Bacterial culture and antimicrobial susceptibility results from bovine milk samples submitted to four veterinary diagnostic laboratories in Australia from 2015 to 2019
Source: Front Vet Sci. 2023 Aug 10;10:1232048. doi: 10.3389/fvets.2023.1232048 (PMC10450625; doi:10.3389/fvets.2023.1232048)
Supplement: Supplementary file 1 [file Table_1.pdf]

Supplementary Table 1: Results of the univariate logistic regression for *Streptococcus uberis* isolates from milk samples submitted to four veterinary diagnostic laboratories in Australia from 2015 to 2019.

Predictor	Category	Positive % (n)	Negative % (n)	OR (95% CI)	P value
Season	Winter	19.9 (892)	20.3 (1318)	Reference	0.034
	Spring	25.6 (1146)	26.9 (1749)	1.0 (0.9-1.1)	
	Summer	27.1 (1216)	27.8 (1810)	1.0 (0.9-1.1)	
	Autumn	27.4 (1229)	25.0 (1622)	1.1 (1.0-1.3)	
Dairy region	Dairy NSW	2.4 (109)	6.8 (444)	Reference	<0.0001
	Dairy SA	2.8 (125)	4.3 (282)	1.8 (1.3-2.4)	
	Gippsland	30.5 (1366)	26.3 (1709)	3.3 (2.6-4.1)	
	Murray Dairy	33.8 (1514)	28.2 (1834)	3.4 (2.7-4.2)	
	Subtropical	3.1(139)	11.3 (735)	0.8 (0.6-1.0)	
	West Vic	27.4 (1230)	23.0 (1495)	3.4 (2.7-4.2)	
Year	2015	25.0 (1122)	24.9 (1619)	Reference	<0.0001
	2016	16.9 (759)	21.3 (1381)	0.8 (0.7- 0.9)	
	2017	30.2 (1352)	30.3 (1967)	1.0 (0.9- 1.1)	
	2018	20.5 (921)	15.3 (996)	1.3 (1.2- 1.5)	
	2019	7.3 (329)	8.2 (536)	0.9 (0.8-1.0)	

Supplementary Table 2: Results of the univariate logistic regression for *Staphylococcus aureus* isolates from milk samples submitted to four veterinary diagnostic laboratories in Australia from 2015 to 2019.

Predictors	Category	Positive % (n)	Negative % (n)	OR (95% CI)	P value
Season	Winter	18.6 (485)	20.6 (1725)	Reference	0.0621
	Spring	27.6 (719)	26.0 (2176)	1.2 (1.0 -1.3)	
	Summer	28.5 (741)	27.3 (2285)	1.2 (1.0- 1.3)	
	Autumn	25.3 (660)	26.1 (2191)	1.1 (0.9- 1.2)	
Dairy region	Dairy NSW	2.3 (60)	5.9 (493)	Reference	<0.0001
	Dairy SA	5.0 (129)	3.3 (278)	3.8 (2.7- 5.4)	
	Gippsland	31.7 (825)	26.9 (2250)	3.0 (2.3- 4.0)	
	Murray Dairy	26.5 (690)	31.7 (2658)	2.1 (1.6- 2.8)	
	Subtropical	8.2 (215)	7.9 (659)	2.7 (2.0- 3.7)	
	West Vic	26.3 (686)	24.3 (2039)	2.8 (2.1- 3.7)	
Year	2015	25.4 (662)	24.8 (2079)	Reference	0.285
	2016	19.6 (512)	19.4 (1628)	1.0 (0.9-1.1)	
	2017	28.6 (745)	30.7 (2574)	0.9 (0.8-1.0)	
	2018	18.4 (479)	17.2 (1438)	1.0 (0.9-1.2)	
	2019	8.0 (207)	7.9 (658)	1.0 (0.8-1.2)	

Supplementary Table 3: Results of the univariate logistic regression for *Escherichia coli* isolates from milk samples submitted to four veterinary diagnostic laboratories in Australia from 2015 to 2019.

Predictors	Category	Positive % (n)	Positive % (n)	OR (95% CI)	P value
Season	Winter	23.7 (209)	19.8 (2001)	Reference	
	Spring	29.8 (262)	26.1 (2633)	1.0 (0.8-1.2)	0.0001
	Summer	25.6 (225)	27.7 (2801)	0.8 (0.6-0.9)	
	Autumn	20.9 (184)	26.4 (2667)	0.7 (0.5-0.8)	
Dairy region	Dairy NSW	6.4 (56)	4.9 (497)	Reference	
	Dairy SA	2.5 (22)	3.8 (385)	0.5 (0.3-0.8)	
	Gippsland	17.9 (158)	28.9 (2917)	0.5 (0.3-0.7)	<0.0001
	Murray Dairy	32.3 (284)	30.3 (3064)	0.8 (0.6-1.1)	
	Subtropical	14.3 (126)	7.4 (748)	1.5 (1.1-2.1)	
	West Vic	26.6(234)	24.7 (2491)	0.8 (0.6-1.1)	
Year	2015	27.1 (239)	24.8 (2502)	Reference	
	2016	19.1 (168)	19.5 (1972)	0.9 (0.7-1.1)	
	2017	26.5 (233)	30.5 (3086)	0.8 (0.7-1.0)	<0.0001
	2018	15.0 (132)	17.7 (1785)	0.8 (0.6-1.0)	
	2019	12.3 (108)	7.5 (757)	1.5 (1.2-1.9)	

Supplementary Table 4: Results of the univariate logistic regression for *Streptococcus dysgalactiae* isolates from milk samples submitted to four veterinary diagnostic laboratories in Australia from 2015 to 2019.

Predictor	Category	Positive<br>% (n)	Negative<br>% (n)	OR (95% CI)	P value
Season	Winter	21.1 (155)	20.1 (2055)	Reference	
	Spring	43.4 (319)	25.1 (2576)	1.6 (1.3-2.0)	<0.0001
	Summer	20.4 (150)	28.1 (2876)	0.7 (0.5-0.9)	
	Autumn	15.1 (111)	26.7 (2740)	0.5 (0.4-0.7)	
Dairy region	Dairy NSW	2.3 (17)	5.2 (536)	Reference	
	Dairy SA	3.8 (28)	3.7 (379)	2.3 (1.3-4.3)	
	Gippsland	29.0 (213)	27.9 (2862)	2.3 (1.4-3.9)	<0.0001
	Murray Dairy	24.6 (181)	30.9 (3167)	1.8 (1.1-3.0)	
	Subtropical	6.0 (44)	8.1(830)	1.7 (0.9-3.0)	
	West Vic	34.3 (252)	24.1 (2473)	3.2 (1.9-5.3)	
Year	2015	17.0 (125)	25.5 (2616)	Reference	
	2016	25.8 (190)	19.0 (1950)	2.0 (1.6-2.6)	
	2017	39.5(290)	29.6 (3029)	2.0 (1.6- 2.5)	<0.0001
	2018	10.1 (74)	18.0 (1843)	0.8 (0.6-1.1)	
	2019	7.6 (56)	7.9 (809)	1.4 (1.0-2.0)	

Supplementary Table 5: Results of the univariate logistic regression for *Streptococcus agalactiae* isolates from milk samples submitted to four veterinary diagnostic laboratories in Australia from 2015 to 2019.

Predictors	Category	Positive % (n)	Negative % (n)	OR (95% CI)	P value
Season	Winter	34.1 (110)	19.7 (2100)	Reference	<0.0001
	Spring	15.5 (50)	26.7 (2845)	0.3 (0.2-0.5)	
	Summer	26.0 (84)	27.6 (2942)	0.5 (0.4-0.7)	
	Autumn	24.4 (79)	26.0 (2772)	0.5 (0.4-0.7)	
Dairy region	Dairy NSW	1.2 (4)	5.1 (549)	Reference	<0.0001
	Dairy SA	18.3 (59)	3.3 (348)	23.3 (8.4-64.6)	
	Gippsland	11.5(37)	28.5 (3038)	1.7 (0.6-4.7)	
	Murray Dairy	46.4 (150)	30.0 (3198)	6.4 (2.4-17.4)	
	Subtropical	19.2 (62)	7.6 (812)	10.5 (3.8-29.0)	
	West Vic	3.4 (11)	25.5 (2714)	0.6 (0.2-1.8)	
Year	2015	18.9 (61)	25.2 (2680)	Reference	<0.0001
	2016	8.7 (28)	19.8 (2112)	0.6 (0.4-0.9)	
	2017	50.1 (162)	29.6 (3157)	2.3 (1.7-3.0)	
	2018	19.2 (62)	17.4 (1855)	1.5 (1.0-2.1)	
	2019	3.1 (10)	8.0 (855)	0.5 (0.3-1.0)	

Supplementary Table 6: Results of the univariate logistic regression for *Corynebacterium bovis* isolates from milk samples submitted to four veterinary diagnostic laboratories in Australia from 2015 to 2019.

Predictors	Category	Positive<br>% (n)	Negative<br>% (n)	OR (95% CI)	P value
Season	Winter	14.9 (94)	20.5 (2116)	Reference	
	Spring	14.1 (89)	27.1 (2806)	0.7 (0.5-1.0)	
	Summer	34.8 (220)	27.1 (2806)	1.8 (1.4-2.3)	<0.0001
	Autumn	36.2 (229)	25.3 (2622)	2.0 (1.5-2.5)	
Dairy region	Dairy NSW	10.8 (68)	4.7 (485)	Reference	
	Dairy SA	0.2 (1)	3.9 (406)	0.0 (0.0-0.1)	
	Gippsland	26.7 (169)	28.1(2906)	0.4 (0.3-0.6)	
	Murray Dairy	34.8 (220)	30.2 (3128)	0.5 (0.4-0.7)	<0.0001
	Subtropical	10.6 (67)	7.8 (807)	0.6 (0.4-0.8)	
	West Vic	16.9 (107)	25.3 (2618)	0.3(0.2-0.4)	
Year	2015	34.0 (215)	24.4 (2526)	Reference	
	2016	14.2 (90)	19.8 (2050)	0.5 (0.4-0.7)	
	2017	31.0 (196)	30.2 (3123)	0.7 (0.6-0.9)	<0.0001
	2018	14.3 (90)	17.7 (1827)	0.6 (0.4-0.7)	
	2019	6.5 (41)	7.9 (824)	0.6 (0.4-0.8)	

Supplementary Table 7: Results of the univariate logistic regression for *Nocardia* spp. isolates from milk samples submitted to four veterinary diagnostic laboratories in Australia from 2015 to 2019.

Predictors	Category	Positive % (n)	Negative % (n)	OR (95% CI)	P value
Season	Winter	14.1 (40)	20.3 (2170)	Reference	<0.0001
	Spring	36.9 (105)	26.1 (2790)	2.0 (1.4-3.0)	
	Summer	30.3 (86)	27.5 (2940)	1.6 (1.1-2.3)	
	Autumn	18.7 (53)	26.1 (2798)	1.0 (0.7-1.6)	
Dairy Region	Dairy NSW	0.7 (2)	5.2 (551)	Reference	<0.0001
	Dairy SA	0.7 (2)	3.8 (405)	1.4 (0.2-9.7)	
	Gippsland	56.7 (161)	27.2 (2914)	15.2 (3.8-61.6)	
	Murray Dairy	16.9 (48)	30.9 (3300)	4.0 (1.0-16.5)	
	Subtropical	4.9 (14)	8.0 (860)	4.5 (1.0-19.8)	
	West Vic	20.1 (57)	24.9 (2668)	5.9 (1.4- 24.2)	
Year	2015	16.2 (46)	25.2 (2695)	Reference	<0.0001
	2016	31.7 (90)	19.2 (2050)	2.6 (1.8-3.7)	
	2017	29.2 (83)	30.2 (3236)	1.5 (1.0- 2.2)	
	2018	13.7 (39)	17.6 (1878)	1.2 (0.8-1.9)	
	2019	9.2 (26)	7.8 (839)	1.8 (1.1-3.0)	

Supplementary Table 8: Results of the intraclass correlation (ICC) one way random-effects model to assess correlation between pathogen occurrence and dairy farm postcode for the seven most common pathogens.

Isolate	ICC (Individual measurement)	95% CI		P value
Staphylococcus aureus	0.42	0.32	0.51	<0.0001
Escherichia coli	0.04	-0.07	0.15	0.227
Streptococcus uberis	0.88	0.86	0.91	<0.0001
Streptococcus dysgalactiae	0.05	-0.06	0.16	0.176
Streptococcus agalactiae	0.00	-0.11	0.11	0.486
Corynebacterium bovis	0.05	-0.06	0.16	0.177
Nocardia spp.	-0.05	-0.16	0.06	0.820
